# Epoch and accuracy based empirical study for cardiac MRI segmentation using deep learning technique

**DOI:** 10.7717/peerj.14939

**Published:** 2023-03-22

**Authors:** Niharika Das, Sujoy Das

**Affiliations:** Maulana Azad National Institute of Technology, Bhopal, India

**Keywords:** Deep learning, Medical image segmentation, Neural network, Convolution networks

## Abstract

Cardiac magnetic resonance imaging (CMRI) is a non-invasive imaging technique to analyse the structure and function of the heart. It was enhanced considerably over several years to deliver functional information for diagnosing and managing cardiovascular disease. CMRI image delivers non-invasive, clear access to the heart and great vessels. The segmentation of CMRI provides quantification parameters such as myocardial viability, ejection fraction, cardiac chamber volume, and morphological details. In general, experts interpret the CMR images by delineating the images manually. The manual segmentation process is time-consuming, and it has been observed that the final observation varied with the opinion of the different experts. Convolution neural network is a new-age technology that provides impressive results compared to manual ones. In this study convolution neural network model is used for the segmentation task. The neural network parameters have been optimized to perform on the novel data set for accurate predictions. With other parameters, epochs play an essential role in training the network, as the network should not be under-fitted or over-fitted. The relationship between the hyperparameter epoch and accuracy is established in the model. The model delivers the accuracy of 0.88 in terms of the IoU coefficient.

## Introduction

Cardiac magnetic resonance imaging is one of the dominant methods to diagnose the condition of patients (Bai et al., 2018). Researchers have developed various methods to perform the segmentation of cardiac MR images. Some methods are semi-automatic, whereas other methods are fully automatic. Various parameters, for example, ejection fraction, ventricular volume, mass, wall thickness, motion abnormality, *etc.*, can be calculated using the segmented cardiac magnetic resonance images (Soomro et al., 2017). Deep learning techniques have drawn significant attention for the segmentation of cardiac MR images (Patravali, Jain & Chilamkurthy, 2017). These techniques are becoming more popular due to their ability to extract hidden features based on the application’s needs, which were impossible to extract with the conventional methods (Baumgartner et al., 2017). The outcomes provided by these new-age technologies are considered valuable and accurate and help experts plan further action for patient treatment and follow-up (Ashraf, 2021). Convolutional networks provide outstanding results for classification-based problems where only one class label exists for an input image. Many classification applications require the labelling of each pixel; as in the case of biomedical imaging, a class label for each pixel is essential. Some convolution neural networks include UNET and recurrent neural networks. Ronneberger, Fischer & Brox (2015) proposed UNET, in which a convolution network was used as a foundation for biomedical image segmentation. The study also suggested that the model can be modified for other biomedical image segmentation requirements. In biomedical images, it is essential to focus on the irregular area to make it easy to predict and detect disease (Ronneberger, Fischer & Brox, 2015). UNET is a symmetric neural network composed of upsampling and down-sampling paths. The accuracy of this type of model heavily depends on the image resolution (Lau et al., 2021), learning parameters, hyperparameters, numbers of hidden layers, data augmentation, optimization techniques, loss function and kind of activation function used *etc.* (Afaq & Rao, 2020). Generally, learning parameters consist of weights and biases. These are called learning parameters because these parameters are initialized with a random value and are adjusted toward values indicating correct output. Hyperparameters are the parameters whose values are initialized once and are constant for the entire training, like epoch, dropout *etc.* Epoch signifies one pass over the entire dataset. The weights are loaded when the training starts and changes when the next epoch completes over the same dataset.

One of the significant challenges associated with these models is choosing the optimal number of epochs. When a model is overfitted, it will also learn the unwanted noise, and the under-fitted model will not learn completely (Afaq & Rao, 2020). Hence, Epoch optimization for over-fitting and under-fitting in the model needs to be established. In biomedical imaging, assigning a class label to each pixel is essential or calculated for training and validation datasets. The training is stopped when the validation error is minimum. The validation set helps in minimizing overfitting. Therefore, the motivation of this paper is to observe the impact of epochs on UNET for the ACDC (Bernard et al., 2018) dataset for cardiac MRI segmentation. For the same, automatic cardiac magnetic resonance image segmentation of left and right ventricles is accomplished using UNET. ACDC Challenge 2017 dataset is used for the training and testing of the network, which consists of data from 100 subjects, including the end systole and end diastole phases. The model is trained and tested for 50 epochs, 100 epochs, 150 epochs and 200 epochs. Finally, the intersection over union (IoU) coefficients are calculated for different epochs and compared.

## Related Work

Ronneberger, Fischer & Brox (2015) presented a “fully convolution network” architecture consisting of contraction and expanding paths. The contraction path mainly focused on capturing the context, whereas the symmetric expanding path was used for precise localization. This architecture could be used for end-to-end training. It was designed in such a way that it could work well with only a few numbers of training data as medical data as annotations acquisition is expensive (Zhang et al., 0000), making it more suitable for medical image segmentation. Afaq & Rao (2020) tried to find the optimal number of epochs for training the network. The authors used two datasets for the experiment. They trained the network for different epochs for both datasets. They also tried to establish a relationship between training and validation errors. Siddique et al. (2018) used a neural network for the classification of handwritten digits of the MNIST dataset. They have made a variation in epochs, hidden layers and batch size to achieve maximum accuracy. With 50 epochs and four hidden layers, the model got the maximum accuracy. For 20 epochs, the model achieved maximum accuracy when there were only three hidden layers. When the number of hidden layers was increased, it was observed that the accuracy dropped. Also, with the increase of hidden layers, the computation complexity increased. Overall, the model achieved the highest accuracy of 97.32%, with four hidden layers, 50 batch sizes and 50 epochs. In Sinha et al. (2010), the authors presented a method to determine the ideal number of epochs with the help of self-organizing maps. They tested their model from 500 to 1,200 epochs and discovered that it delivered the best results around 1,100 epochs. While training the backpropagation network, the model got the least absolute error with around 1,100 epochs. When the value of epochs was increased or decreased, the model generated more errors. A deep learning based 3D myocardial segmentation method was proposed (Curiale et al., 2017), which used Jaccard distance as objective loss function, batch normalization and residual learning strategies to increase segmentation precision. A quality control-driven (QCD) segmentation framework was proposed (Hann et al., 2021) for image analysis and quality control. Based on the prediction of segmentation accuracy, optimal segmentation was chosen on-the-fly. A comparison was made between 2.5D methods against 2D and 3D CNN, including various imaging modalities and regions. The authors (Zhang et al., 0000) intended to investigate the 2.5D method to overcome the lack of volumetric information of 2D methods and the massive computational cost of 3D methods. A deep learning algorithm incorporated with a deformable model for automatic segmentation of LV was proposed (Avendi, Kheradvar & Jafarkhani, 2016). The algorithm learned the segmentation task from ground truth and detected the LV. The inferred LV shape received from the stacked autoencoders was passed to the deformable models to improve the accuracy of the overall system.

## Methodology

The segmentation of the left ventricle and right ventricle is accomplished with the help of the convolution neural network technique. Cardiac magnetic resonance imaging datasets from the ACDC challenge (Bernard et al., 2018) are applied here for training, validation and testing. Pre-processing of data is performed to bring down the problem into a single-channel problem.

### Dataset

For the experiment purpose, the cardiac magnetic resonance image dataset of the ACDC challenge (Bernard et al., 2018) is used. The dataset includes images of 100 patients in short-axis cine-MRI captured on 1.5T and 3T systems and has resolutions ranging from 0.70 × 0.70 mm to 1.92 × 1.92 mm in-plane and 5 mm to 10 mm through-plane. Along with the original images, ground truth for every patient is provided for the left ventricle (LV), the myocardium (Myo), and the right ventricle (RV). The data is available for both end-diastole (ED) and end-systole (ES) phases. The entire dataset is parted into five groups. Each group comprise data of twenty patients. These groups are divided by the heart condition of patients. The group contain data from normal patients (NOR), patients with systolic heart failure with infarction (MINF), patients with dilated cardiomyopathy (DCM), patients with hypertrophic cardiomyopathy (HCM) and patients with abnormal right ventricles (ARV). The dataset is split arbitrarily for training, validation and testing purposes. The training data from 70 patients are used; for validation data from 10 subjects is used; for the testing, data from 20 subjects is used. To be more precise, a total of 1,328 sets of input and label images are used for training, 177 sets of input and label images are used for validation, and 366 sets of input and label images are used for testing. The availability of ground truth for both left and right ventricles is the main inspiration for choosing the ACDC dataset.

### Preprocessing

The dataset is initially stored in NIFTI (Cox et al., 2004) format. The images from the dataset are converted to portable network graphics (PNG) format. Also, the images are of various resolutions; for example, some have 216 × 256 size, 222 × 224 size, 208 × 256 size *etc.* Thambawita et al. (2021) discussed the performance of CNN with different resolutions. They found that maximum accuracy was achieved with higher resolution. Therefore, in this study nearest higher resolution is chosen. All the images are resized to a universal resolution of 256 × 256. Considering the GPU memory limitation of the machine on which experiments are performed, images with higher resolution are not feasible. The segmentation problem is also converted to a single-class problem. To convert this problem into a single-channel problem, pixel values of the left ventricle and right ventricle are kept the same in the label images. No alteration was made in the pixel values of the myocardium in the labelled images.

### Network architecture

The UNET architecture is the inspiration behind this network architecture (Ronneberger, Fischer & Brox, 2015). As implied by the classic UNET architecture, this architecture also has a U-like shape in the form of a Contracting path and an expensive one. The size of the input image is 256 × 256. In the contracting path, the following blocks are arranged five times. Each block has two convolutional blocks and two batch normalization layers. Both layers are succeeded by the rectified linear unit (ReLU) activation function. The max-pooling operation follows the activation function. The patch size in the max pooling operation is 2 × 2. The value of padding is set to “same”.

The dropout value is set to 0.3. The Dropout layers are applied to minimize the effect of overfitting. When dropout is used, weights of randomly selected networks are neglected for activation on the next forward pass. Likewise, during the backward pass, the weight of selected neurons is not updated. When the weights of randomly chosen neurons are dropped out, other neurons that are not dropped out predict the weights on behalf of absent neurons. The dropout is used to increase the generalization efficiently by reducing the influence of the weights of neurons. Max pooling is also combined following every convolution layer. Max pooling assists in decreasing the resolution of the image so that a greater fragment of an image can be evaluated at one time. It supports the network to decrease the total parameters and eventually reduce the computation time. Batch normalization is a technique to normalize the inputs to a layer in the network. Further, batch normalization supports providing some stability in the learning process. This eventually reduces the training epochs to train the network entirely. The ReLU activation function is applied in this model. The main reason for applying the ReLU activation function is that it is simple to compute and adds a negligible burden on the machine.

The ReLU activation function does not activate all the neurons simultaneously by taking the benefits of sparsity. The function can be represented as follows:

\begin{eqnarray*}f(u)=max(0,u). \end{eqnarray*}

**Figure 1:**
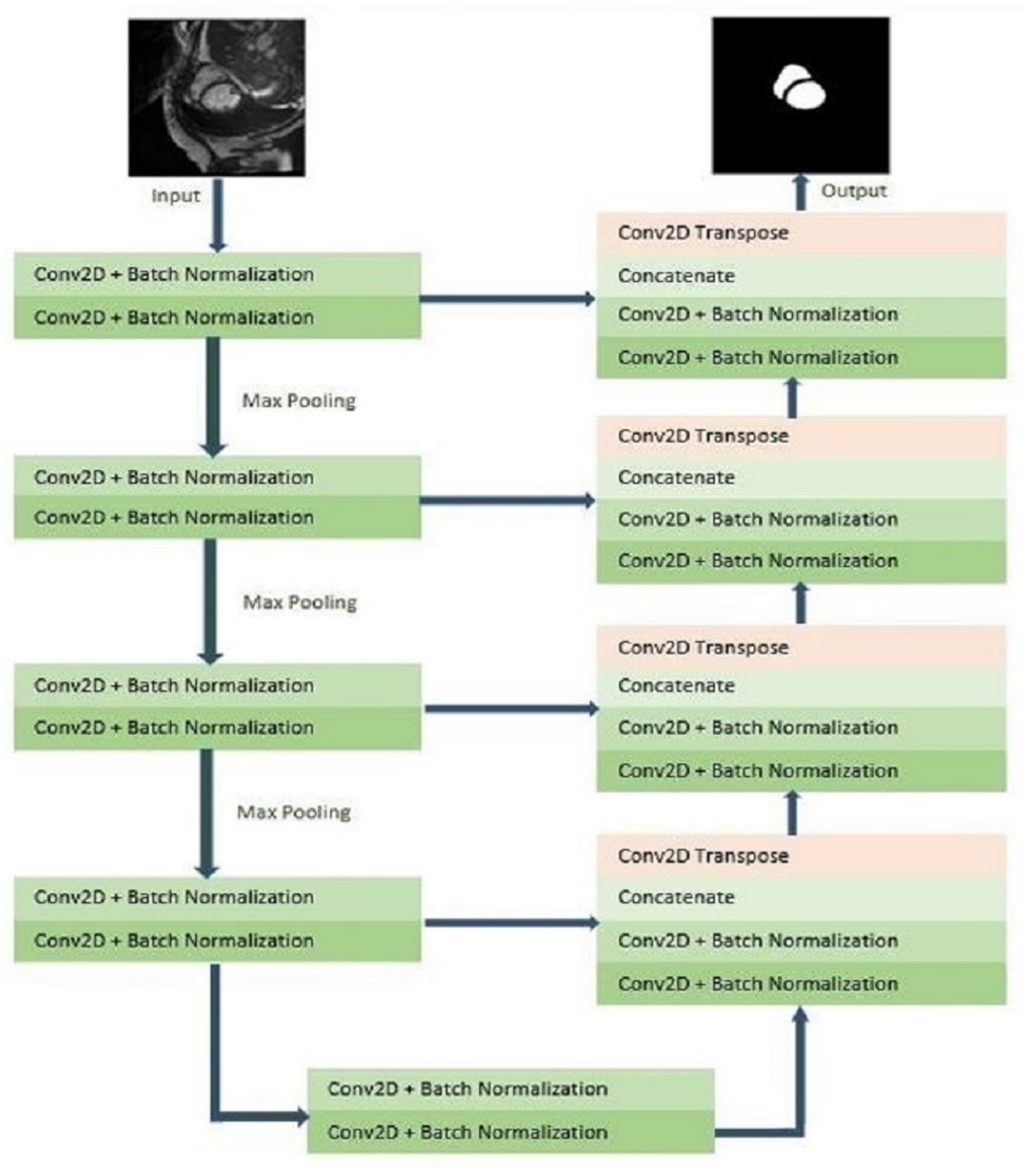
2D model of CNN architecture.

The ReLU function is monotonic. For any negative input, it returns 0, which means the neurons do not get activated. For any positive value u, it simply returns that value. The output may vary in the range from 0 to infinity. The main reason for using this activation function is that it converges very fast. The number of feature channels is doubled at every downsampling step. In the expensive path, transposed convolution and concatenation layer is added with the two convolutional and batch normalization layers. During the process, the number of feature channels is made half by the transposed convolution. In the expensive path, rectified linear unit (ReLU) activation function is used at four layers. In the output layer, the sigmoid activation function is used. Here too, the value of padding is set to “same”. The network architecture is shown in Fig. 1.

### Training

Original images of cardiac MRI segmentation and their corresponding ground truth are used for the training. Overall, 1,328 pairs of images and labels are used for training the network, and 172 image and label pairs are used for validation purposes. HP OMEN series personal computer with Intel^®^ Core™ i9-9880H CPU 2.30 GHz, 2,304 Mhz, 8 Cores, with 32 GB RAM and NVIDIA GeForce RTX 2080 is used for the training purpose. The training is performed for 50 epochs, 100 epochs, 150 epochs and 200 epochs. It took around 25 hours, 50 hours, 75 hours and 100 hours to complete the training for 50 epochs, 100 epochs, 150 epochs and 200 epochs, respectively. The total number of parameters is 34,535,810; among them, there are 34,524,034 trainable and 11,776 non-trainable parameters. The main difference between trainable and non-trainable parameters is that the values of trainable parameters are updated according to their gradient, but the values of non-trainable parameters are not updated as per their gradients. The weights of non-trainable parameters cannot be modified using backpropagation.

### Optimization function

For the experiment purpose, given optimization functions are used:

### Categorical cross-entropy loss

Categorical cross-entropy loss is one of the universal methods used for measuring loss. In multi-class classification, the number of classes can be multiple. However, the object can belong to only one class. Based on the output provided by the model, it is decided to which class the object will belong. The categorical cross-entropy evaluates the difference between two probability distributions (https://towardsdatascience.com/cross-entropy-loss-function-f38c4ec8643e). The loss value establishes the match between the object’s predicted class and the object’s actual class.

The categorical cross-entropy loss function is calculated as follows:

\begin{eqnarray*}\text{Loss}=-\sum _{\mathrm{i}=1}^{\text{Output size}}\mathrm{Ai}.\log \nolimits \mathrm{\^{A} i} \end{eqnarray*}

where Âi is the i-th scalar value in the model output, A_i_ is the parallel target value, the output size is the number of scalar values in the model output.

### IoU coefficient and loss

The intersection over u, also known as the Jaccard index (Real & Vargas, 1996), is one of the famous metrics used to evaluate the similarity between the segmented image and the ground truth of the corresponding image. The IoU coefficient is calculated by the area of overlap between predicted values and the ground truth divided by the union of predicted values and the ground truth. In other words, it can be said that IoU metrics compute the number of pixels common between prediction and ground truth divided by the total number of pixels existing around both masks. Here, I denotes the region encircled by the segmentation algorithm, and P denotes the region encircled by the ground truth. The value of the IoU coefficient can be determined as follows:

\begin{eqnarray*}IoU(I,P)={|}I\cap P{|}/{|}I\cup P{|}. \end{eqnarray*}

The range of IoU can vary from 0 to 1. A greater value of IoU indicates higher accuracy, *i.e.,* 0 represents the absolute mismatch, whereas 1 represents a perfect match. Similarly, 1- IoU can be used to calculate the loss to maximize the overlap between two images. *i.e.,*

\begin{eqnarray*}Loss=1-({|}I\cap P{|}/{|}I\cup P{|}). \end{eqnarray*}

More precisely, loss evaluates the loss of information locally and globally. The loss is an important parameter to measure accuracy.

**Figure 2:**
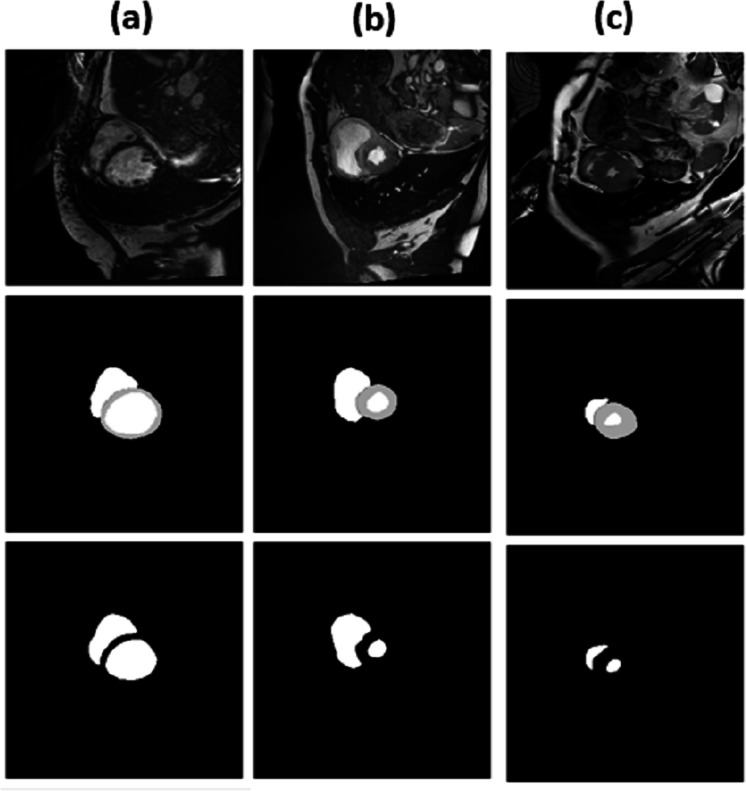
(A–C) Results from epoch 50.

**Figure 3:**
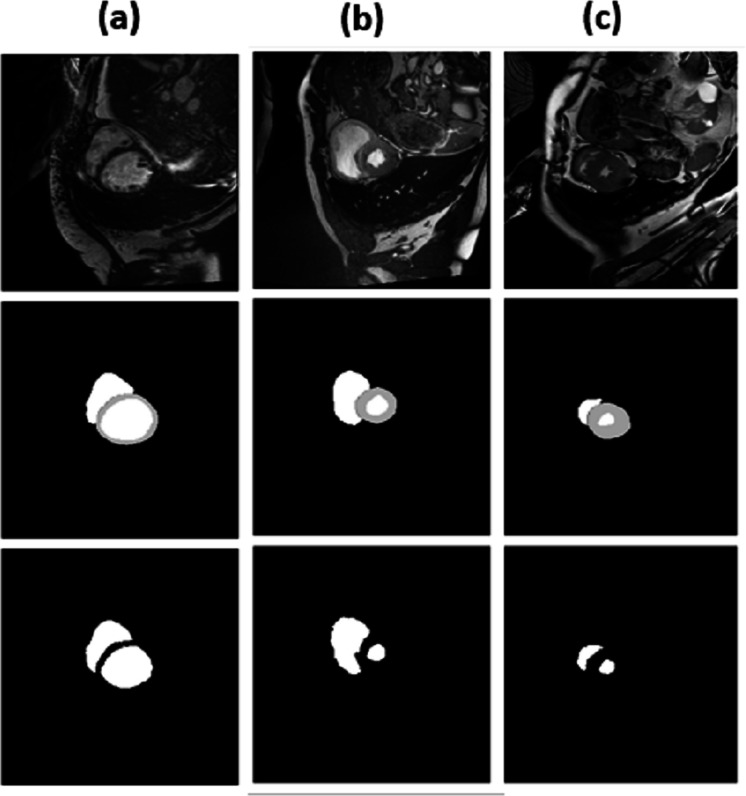
(A–C) Results from epoch 100.

**Figure 4:**
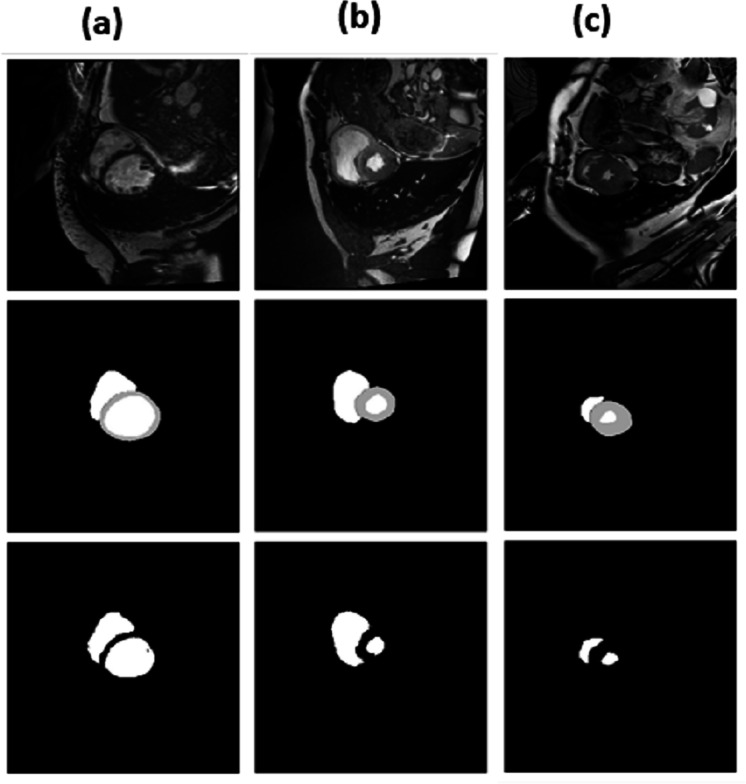
(A–C) Results from epoch 150.

**Figure 5:**
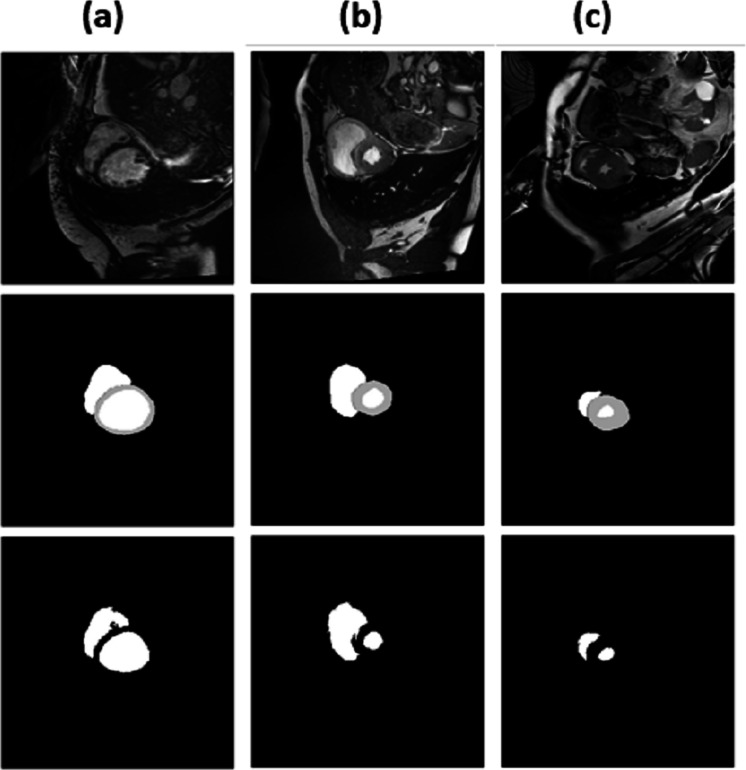
(A–C) Results from epoch 200.

**Figure 6:**
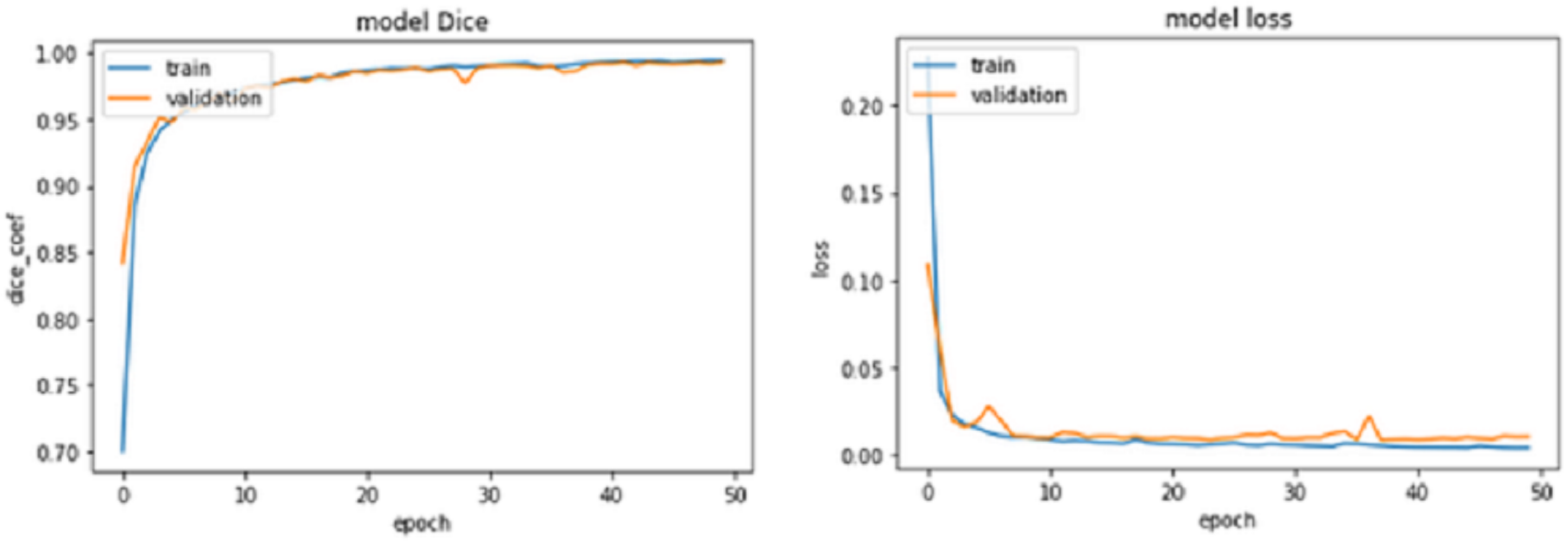
IoU Coef for train and validation data sets for 50 epochs.

**Figure 7:**
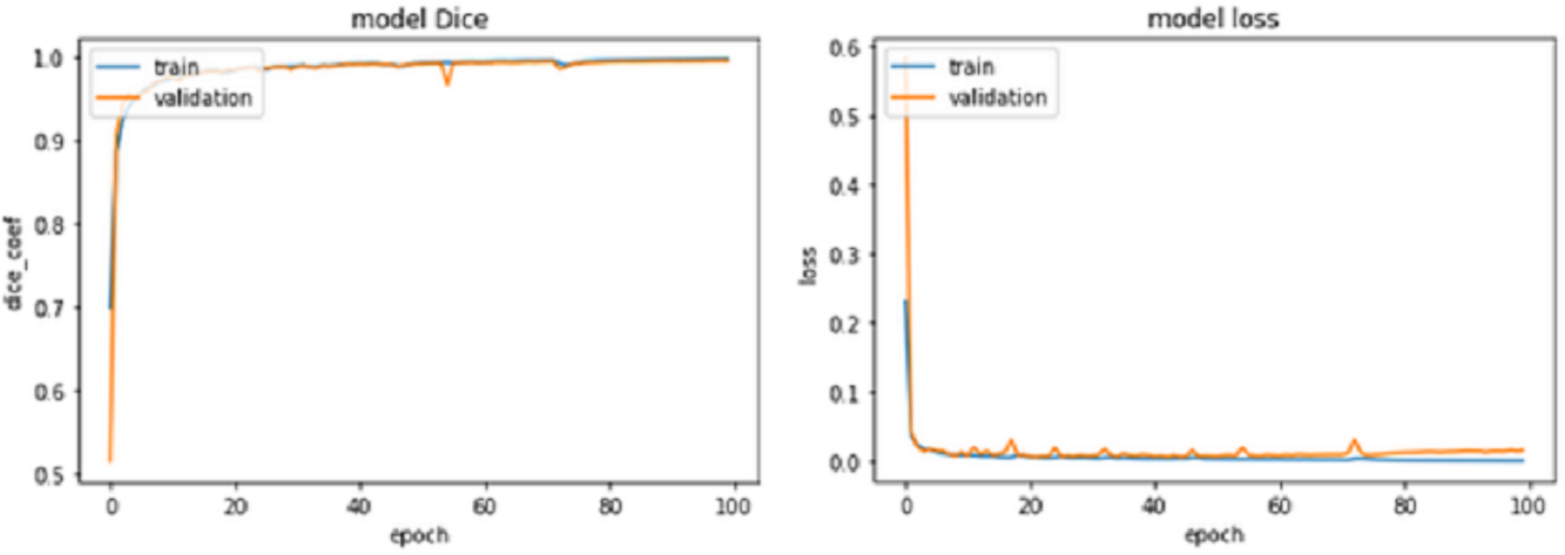
IoU Coef for train and validation data sets for 100 epochs.

**Figure 8:**
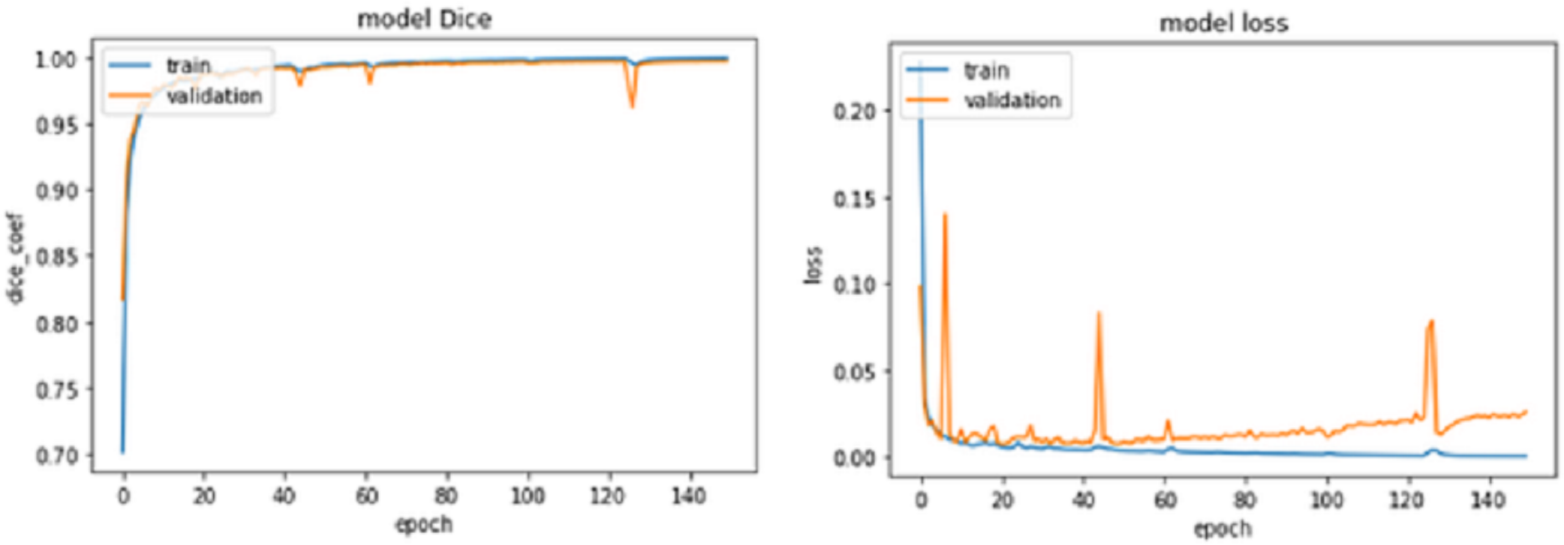
IoU Coef for train and validation data sets for 150 epochs.

**Figure 9:**
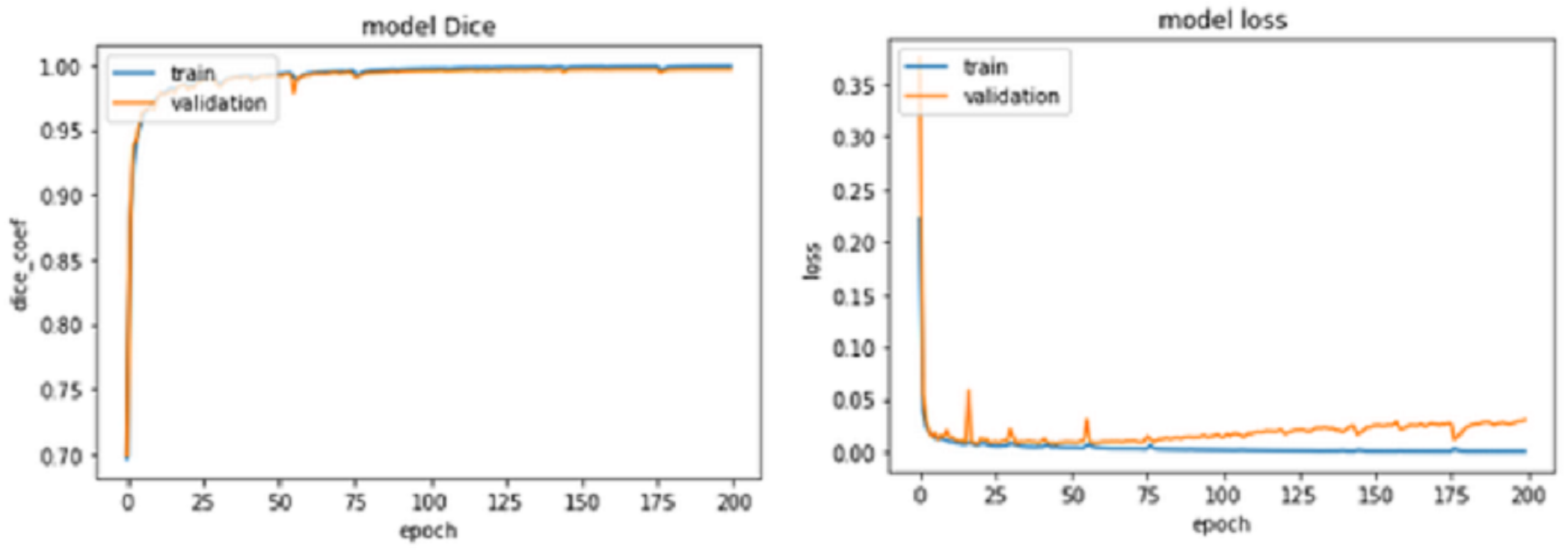
IoU Coef for train and validation data sets for 200 epochs.

**Table 1:** Quantitative evaluation of the model for various epochs.

**No. of epochs**	**No. of Images for training**	**No. of Images for Validation**	**No. of Images for Testing**	**IoU_Coef. for training set**	**Loss for training set**	**Accuracy for training set**	**IoU_Coef. for Validation set**	**Loss for Validation set**	**Accuracy for Validation set**	**Training Time (Approx.)**	**Iou Accuracy for testing set**	**Variance**	**Testing time**
50	1,328	172	366	0.9944	0.0034	0.9985	0.9927	0.0102	0.9970	25 h	0.8661	0.1159	154 s
100				0.9979	0.0012	0.9995	0.9959	0.0163	0.9972	50 h	0.8715	0.1119	160 s
150				0.9992	4.2822e−04	0.9998	0.9968	0.0258	0.9972	75 h	0.8852	0.1015	100 s
200				0.9996	1.8292e−04	0.9999	0.9967	0.0303	0.9970	100 h	0.8715	0.1119	107 s

## Results and Discussion

For epochs 50, 100, 150 and 200, miscellaneous statistics are computed for the segmentation results produced by this network architecture. Also, the visual difference between the ground truth and segmentation results is analyzed. Epoch 50 results are shown in Figs. 2A–2C. Epoch 100 results are shown in Figs. 3A–3C. Epoch 150 results are shown in Figs. 4A–4C. Epoch 200 results are shown in Figs. 5A–5C. For epochs 50, 100, 150, and 200, a combined IoU coefficient and loss graphs are shown in Figs. 6–9 for the training and validation phases. The graph shows a significant increase in IoU at the start, but after a couple of epochs, the curve does not show a drastic change. From the graph, it can be interpreted that there is a gradual decrease in loss with the increasing number of training steps, but for epochs 150 and 200, increased values for validation data can be observed in the loss graph. The visual examples of prediction are shown in Figs. 2–5. Images are taken from the basal slices, middle slices, and slices from the apex for the study. It can be interpreted from the visual inspection that the prediction matches the ground truth for the left and right ventricles in almost all cases. For epoch 200, in some cases, over-training of data is observed. The model has achieved a significant IoU score of 0.86, 0.87, 0.88 and 0.87 for 50, 100, 150 and 200 epochs, respectively. The model also achieved variance of 0.11, 0.11, 0.10, and 0.11 for 50, 100, 150 and 200 epochs, respectively. It took 154 s, 160 s, 100 s and 107 s to segment 366 images. Overall, it can be stated that training with 150 epochs provided the best results. Table 1 summarizes the quantitative evaluation for training, validation, and testing sets. Due to the intensity inhomogeneity and fuzzy boundaries near the apex, it isn’t easy to segment the images near it. Despite that, the model performance is remarkable, even in basal and apical slices.

## Conclusion

Deep learning architecture is adapted for the segmentation of the left ventricle and right ventricle of the human heart in the short axis of MRI scans. For the same purpose fully convolution network model is designed and implemented with learning parameters. A total of 1,328 image/label pairs for training, 172 image/label pairs for validation and 366 image/label pairs for testing are used for the algorithm. The model is trained and tested for the 50 epochs, 100 epochs, 150 epochs and 200 epochs. It took 25 h, 50 h, 75 h and 100 h to train the network for 50 epochs, 100 epochs, 150 epochs and 200 epochs, respectively. After training for testing, the algorithm took 154 s, 160 s, 100 s and 107 s to segment 366 images. The algorithm reached an accuracy of 0.88 in terms of the IoU coefficient with 150 epochs.

##  Supplemental Information

10.7717/peerj.14939/supp-1 Supplemental Information 1 Code file Before executing the code one has to update the file location of training, validation and testing data.

## Additional Information and Declarations

### Data Availability

The following information was supplied regarding data availability: The data are available at the Automated Cardiac Diagnosis Challenge: https://acdc.creatis.insa-lyon.fr/.
